# CO_2_ signalling in plants

**DOI:** 10.1098/rstb.2024.0247

**Published:** 2025-05-29

**Authors:** Christine Foyer, Anran Wang, Kai Shi

**Affiliations:** ^1^ School of Biosciences, College of Life and Environmental Sciences, University of Birmingham, Birmingham, UK; ^2^ Department of Horticulture, Zhejiang University, Hangzhou, Zhejiang, People’s Republic of China; ^3^ Hainan Institute, Zhejiang University, Hangzhou, Zhejiang, People’s Republic of China

**Keywords:** carbon dioxide enrichment, stomata, pathogen resistance, reactive oxygen species, systemic signalling, environmental stress

## Abstract

Elevated atmospheric CO_2_ concentrations increase the productivity of plants by enhancing the photosynthesis/photorespiration balance. It has long been recognized that CO_2_ acts as a signal, for example, in the regulation of stomatal closure, as well as the substrate of photosynthesis. Early concepts of CO_2_ signalling in plants focused on a mode of action based on alterations in primary metabolism, particularly sugar availability and signalling. However, stomatal guard cells employ a CO_2_ sensing and signalling mechanism that is independent of sugars. We discuss the possibility that a similar pathway exists in all cells, where it drives calcium-mediated waves of reactive oxygen species (ROS), facilitating cell-to-cell communication of biotic and abiotic threats. The plasma membrane H_2_O_2_ receptor HPCA1 is required for both stomatal closure and the systemic transmission of stress signals. Moreover, increased oxidation of the apoplast activates G protein functions and alters sugar processing and signalling in the apoplast. We discuss the concept elevated CO_2_ constitutes an environmental stress that has a positive effect on plant innate immunity but a negative impact on crop nutritional quality.

This article is part of the theme issue ‘Crops under stress: can we mitigate the impacts of climate change on agriculture and launch the ‘Resilience Revolution’?’.

## 1. Introduction

Average atmospheric CO_2_ levels have increased from 280 μmol mol^−1^ in 1750 to current levels of about 420 ppm, with an increase of about 20% occurring in the last 50 years. While the CO_2_ fertilization effect is beneficial because of increased plant biomass and yield [1,2], this enhancement comes at the cost of nutritional quality [3]. While many crops grown show an increase in yield under elevated CO_2_, the levels of micronutrients (zinc, iron) and proteins will decrease [4–6].

**Figure 1.**
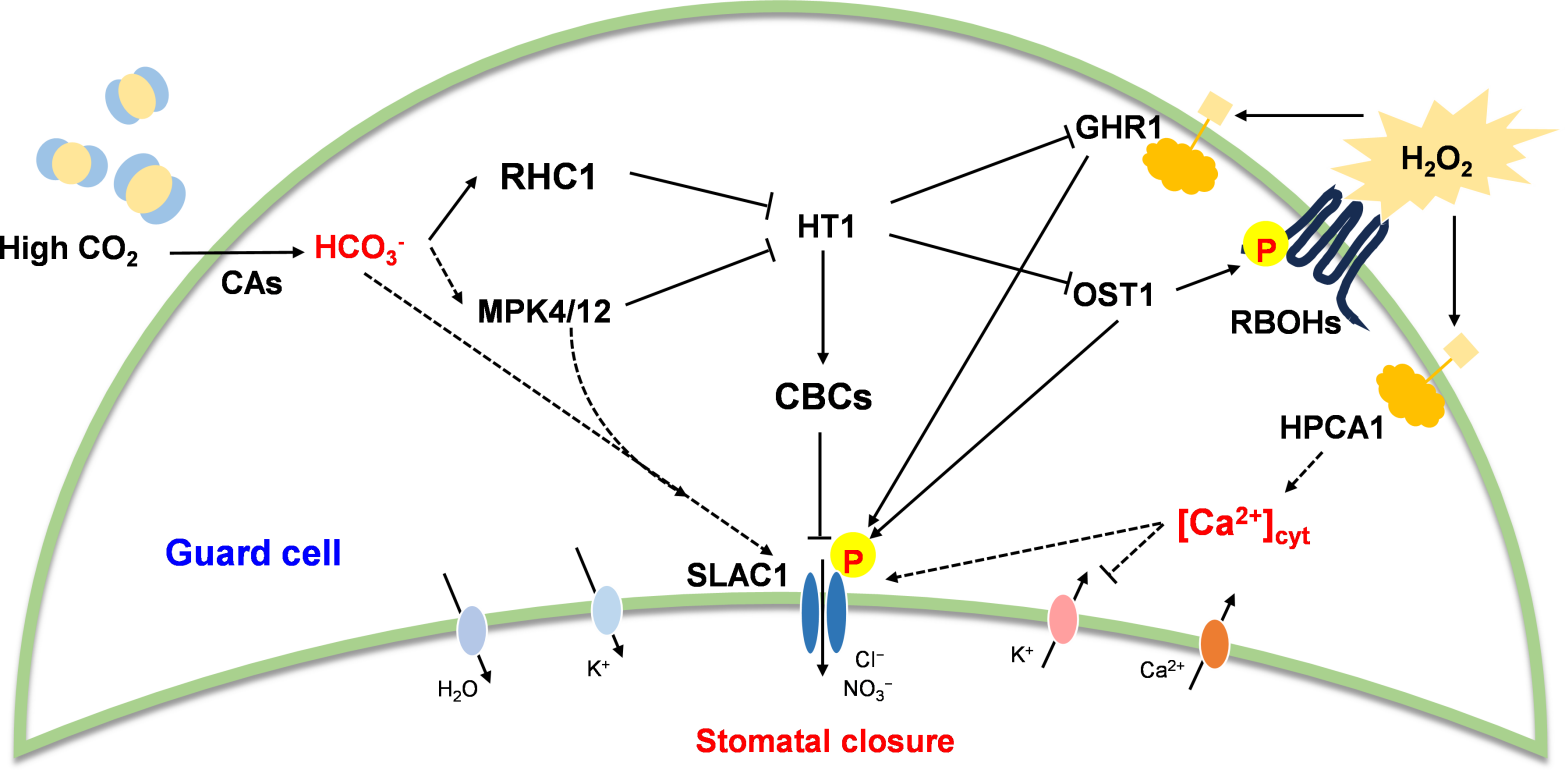
Pathways of CO_2_–dependent stomatal closure in guard cells. CA, carbonic anhydrase; CBC, Convergence of blue light and CO_2_ 1; GHR1, Guard cell hydrogen peroxide resistant, HPCA1, Hydrogen peroxide Ca^2+^ increases 1; HT1, High leaf temperature 1; RBOH, respiratory burst oxidase homolog; ROS, reactive oxygen species; MAPK, Mitogen- activated protein kinase; OST1, Open stomata 1; RCH1, Resistant to high CO_2_ 1; SLAC1, Slow anion channel-associated 1.

Elevated atmospheric CO_2_ levels (eCO_2_) have a direct positive effect on plant growth because of the depression of photorespiration and resultant stimulation of photosynthesis in C_3_ plants [7,8]. Increased CO_2_ assimilation exerts a strong effect on the assimilation of other essential minerals, exerting a strong influence on overall nitrogen status [9]. Higher photosynthesis rates also place considerable demands on other nutrients such as sulphur and phosphorus [10]. Changes in atmospheric CO_2_ levels are sensed by both photosynthesis-dependent and photosynthesis-independent mechanisms. Photosynthesis and associated sugar signalling drive eCO_2_-dependent changes in plant growth, development and defence. However, photosynthesis-independent signalling systems have become of increasing interest in recent years [11]. Of these, CO_2_-dependent regulation of stomatal closure has been well documented [12]. An increasing number of components involved in CO_2_ perception and signalling leading to stomatal closure have been identified (figure 1) including the multidrug and toxic compound extrusion (MATE)-type transporter Resistant to high CO_2_ [13] and two carbonic anhydrases [14].

While little is known about the integration of these pathways with photosynthesis-dependent CO_2_ signalling pathways, the concept that high CO_2_ triggers ROS production in the guard cells via activation of NADPH oxidase/respiratory burst oxidase homolog (RBOH) proteins is generally accepted [15,16]. The RBOH-type NADPH oxidases are largely localized on the plasma membrane, where they generate localized ROS bursts into the apoplast to regulate growth, developmental processes and stress responses [17]. They are also key players in cell-to-cell signalling in response to various stresses and other stimuli [18,19]. The ROS-dependent redox signalling pathways operate together with other central signalling components [20], particularly calcium, G-proteins, hormones, mitogen activated protein (MAP) kinases and reactive nitrogen species, particularly NO and related compounds such as *S*-nitrosoglutathione [21,22], to regulate biotic and abiotic stress responses.

Growth under eCO_2_ increases the levels of endogenous H_2_O_2_ and NO, which are a hallmark of living cells [23–26]. ROS and NO signalling pathways interface with sugar and metabolite signalling. In this context, this review drafts an integrated approach to the CO_2_ perception and signalling pathways in plants, focusing on the dual role of CO_2_ as a carbon source and environmental signal. We address the question of whether cells other than stomatal guard cells independently sense CO_2_, highlighting the evidence that supports the concepts that such sensing occurs in all cells, through similar pathways to those reported in guard cells, and consider how perception of eCO_2_ may function as an environmental signal that amplifies plant innate immune responses.

## 2. CO_2_- dependent regulation of the development and function of stomata

Atmospheric CO_2_ levels are thought to have shaped the size/density relationships of stomata, together with their physiology and regulation. Functioning as regulated epidermal pores on aerial plant organs, stomata are formed by at least a pair of guard cells. These pores facilitate and regulate the exchange of oxygen, CO_2_ and water vapour between plants and the atmosphere, in response to metabolic cues and environmental stimuli. Stomatal functions are vital for essential plant processes, including photosynthesis, respiration, transpiration and temperature control [27]. Crucially, stomata are an integral feature of the plant innate immune system, playing an important role in restricting pathogen invasion [28–30]. Stomatal development is regulated by multiple factors, including CO_2_, ROS, NO and the MAPK pathway. NO and MAPK positively and negatively regulate stomatal development, respectively [31–34]. Atmospheric CO_2_ levels regulate the development and regulation of stomata [35]. The evolutionary adaptation to CO_2_ exerted strong effects on stomatal size (SS) and density (SD). Plants that have adapted to the ‘low’ CO_2_ atmospheres of the past 10 million years showed adjustments of SS and SD, while members of the same species adapted to ‘high’ CO_2_ levels showed no response [36].

The stomatal lineage in *Arabidopsis* begins with the conversion of a subset of protodermal cells into meristemoid mother cells, which then asymmetrically divide to yield a small triangular meristemoid and a large stomatal lineage ground cell (SLGC). After cycles of asymmetric division, the meristemoid differentiates into a guard mother cell, which finally divides symmetrically to form guard cells. NO is known to promote stomatal development [32,33]. Similarly, the pattern of hydrogen peroxide (H_2_O_2_) accumulation in epidermal cells plays a key role in the formation of stomata. H_2_O_2_ is accumulated in meristemoids, which are the stomatal precursors that undergo one to three asymmetric divisions to produce guard mother cells. The master regulator of stomatal development called SPEECHLESS (SPCH) directly binds to the promoters of the *CAT2* and ascorbate peroxidase (*APX)1* genes to repress their expression in meristemoid cells. H_2_O_2_ activates the energy sensor called Sucrose non-fermenting- related kinase (SnRK)1 by inducing the nuclear localization of the catalytic α-subunit KIN10, which stabilizes SPCH to promote stomatal development [37]. Moreover, NO-mediated *S*-nitrosylation of MPK6 at Cys−201 inhibits kinase activity, thereby positively regulating stomatal development and stress responses [38].

**Figure 2.**
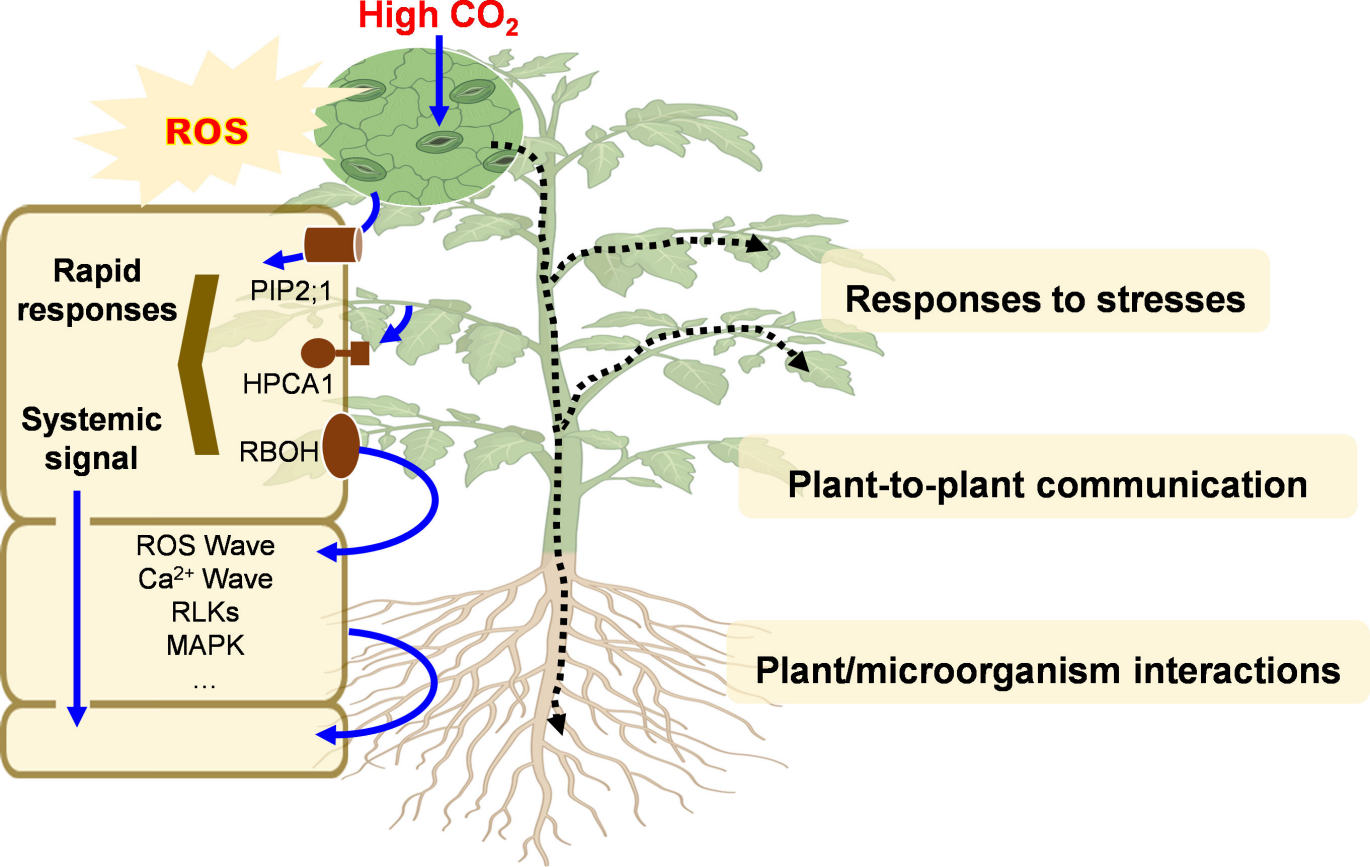
Interactions between ROS and CO_2_ signalling in systemic acclimation responses. HPCA1, Hydrogen peroxide Ca^2+^ Increases 1; RBOH, respiratory burst oxidase homolog; RLK, receptor-like kinases; ROS, reactive oxygen species; MAPK, Mitogen activated protein kinase.

An increasing number of receptors and downstream proteins that participate in the pathways that lead to stomatal closure upon perception of eCO_2_ are being identified (figure 1). For example, ABA-receptors bind to Clade A protein phosphatases type 2C (PP2Cs), which in turn phosphorylate a variety of proteins including SnRK2 proteins. SnRK2s phosphorylate downstream targets including RBOH enzymes. The perception of eCO_2_ leads to RBOH-mediated ROS production and stomatal closure [12]. The Raf-like kinases called CONVERGENCE OF BLUE LIGHT AND CO_2_ (CBC) 1 and CBC2 function as negative regulators of stomatal opening, probably via inhibition of PM H^+^-ATPase activity through C-terminal threonine phosphorylation [39]. The CBC1 kinase is a negative regulator of stomatal closure in response to eCO_2_. Perception of eCO_2_ triggers an interaction between the MAP kinases MPK4/MPK12 and the Raf-like protein kinase called HIGH LEAF TEMPERATURE 1 (HT1) that leads to an inhibition of HT1 kinase activity [40]. Double mutants that are defective in MPK4 and MPK12 show constitutively open stomata. These mutants are sensitive to ABA but are insensitive to eCO_2_ concentrations, suggesting that these MAP kinases are redundant positive regulators of early CO_2_ signal transduction in guard cells [41]. However, MAP kinase activity was not required for CO_2_ sensor function and CO_2_-triggered HT1 inhibition and stomatal closure [40]. Such findings highlight the complexity of CO_2_ perception, which may or may not involve a downstream oxidative burst (figure 2).

## 3. Reactive oxygen species waves and systemic signalling

The plasmalemma-associated RBOH enzymes are the most important sources of ROS in the apoplast and cell wall space [19,42]. Class III heme peroxidases and several other enzymes may also contribute to the accumulation of ROS in the apoplast [43]. The production of ROS by RBOH enzymes is activated by physical and chemical signals and is intrinsically linked to calcium signalling [21]. Changes in the H_2_O_2_ levels in the apoplast of individual cells are sensed by RLKs that trigger an auto-propagating cell-to-cell signalling pathway called the ROS wave (figure 2) that mediates systemic signalling and acclimation responses to the perception of environmental stresses [44]. Changes in apoplastic ROS levels in *Arabidopsis thaliana* are perceived by the cell surface leucine-rich repeat receptor-like kinase (LRR-RK) receptor called Hydrogen peroxide Ca^2+^ increases 1 (HPCA1), which is highly conserved in land plants [45]. HPCA1, which is also called CANNOT RESPOND TO DMBQ1 (CARD1), is activated by H_2_O_2_ via covalent modification of extracellular cysteine residues, to activate a MAP kinase signalling cascade within the cell to induce appropriate stress responses [45]. However, it is interesting to note that MAPK activation following MAMP perception is, in some cases, independent of the activation of NADPH oxidases. For example, this seems to be the case, with regard to β-aminobutyric acid (BABA) priming, which occurs through the malectin-like IMPAIRED OOMYCETE SUSCEPTIBILITY1 (IOS1) LRR-RLK kinase [46]. This kinase, which is critical to pathogen-triggered immunity responses in *Arabidopsis*, forms complexes with BRASSINOSTEROID INSENSITIVE1-ASSOCIATED KINASE1 (BAK1)-dependent cell surface localized pathogen recognition receptors such as FLAGELLIN SENSING2 and the EF-2 receptor EFR and the BAK1-independent CHITIN ELICITOR RECEPTOR KINASE1 (CERK1).

In guard cells, HPCA1/Card1 facilitates the H_2_O_2_-induced activation of calcium channels that is essential for stomatal closure [45]. In addition, DMBQ initiates Ca^2+^ signalling in roots and is important for quinone signalling and the development of the haustoria in plants that parasitize roots [47]. HPCA1/Card1 is required for the cell-to-cell propagation of calcium signals in the transmission of cell-to-cell communication that activates systemic acclimation responses to biotic and abiotic stresses [44]. Diverse stimuli that trigger RBOH-mediated oxidation of the apoplast oxidize the extracellular cysteine residues on HPCA1 that activate the kinase leading to autophosphorylation of amino acid residues in the cytosolic domain that facilitate gating of calcium channels. ROS-wave signalling occurs in animal and plant cells, occurring in cell-to-cell, plant/microorganism [22,48] and plant-to-plant communication [49]. Another receptor-like kinase called GUARD CELL HYDROGEN PEROXIDE RESISTANT (GHR1), described in *A. thaliana*, is also involved in stomatal movement and H_2_O_2_ signalling [50]. The *ghr1* mutants are impaired in stomatal responses to abscisic acid (ABA) and in the H_2_O_2_-regulated activation of S-type anion currents in guard cells. GHR1 catalyzes the phosphorylation of the *S-*type anion channel called SLOW ANION CHANNEL-ASSOCIATED1 (SLAC1) [50]. GHR1 is a receptor-like inactive pseudokinase that facilitates stomatal closure in response to high CO_2_ concentrations and light/dark transitions, as well as apoplastic ROS and ABA. The GHR1-mediated activation of SLAC1 has been suggested to occur via protein/protein interactions involving components such as CALCIUM-DEPENDENT PROTEIN KINASE3 [51].

Within the context of stomatal closure, ROS act as second messengers that decrease guard cell turgor and close the stomatal pores through decreased H^+^ export and K^+^ uptake, while increasing the efflux of K^+^, Cl^−^ and malate. ROS accumulation in intracellular compartments is likely to contribute to the overall regulation of this process [52].

### (a) Guanine nucleotide-binding protein signalling

Signalling through the heterotrimeric guanine nucleotide-binding protein (G protein) complex plays a crucial role in plant development and stress responses [53,54]. The G protein complex consists of three subunits: Gα, Gβ and Gγ. When Gα binds to the guanine nucleotide binding protein (GDPBP), the Gα·GDP complex is able to exchange Guanosine-5'-triphosphate (GTP) for bound GDPBP. The resultant Gα·GTP complex dissociates from the βγ subunits, allowing them to bind to effector molecules [53]. Interactions between G protein and ROS signalling pathways have been documented in plant stress responses. For example, the first phase of the biphasic oxidative burst that is induced by ozone is greatly attenuated or completely absent in *Arabidopsis* mutants lacking Gα protein or Gβ protein. Cytosolic Ca^2+^ levels activate RBOH activity by regulating the interactions between G proteins and the RBOH proteins [36]. In addition, apoplastic ROS activate G protein functions through the Gα subunit, XLG2 [55,56]. The *gpa1* mutants show impaired ABA-induced ROS production, with a consequent inhibition of Ca^2+^- channel activation [57]. Moreover, the Gβ subunit functions together with AtRbohD and AtRbohF to orchestrate disease resistance to *Pseudomonas syringae* that infects plants such as *Arabidopsis* through wounds or stomata [58]. Grain length in rice was found to be regulated by the CC-type glutaredoxin WG1/OsGRX8, which has disulfide oxidoreductase activity. The redox-modulated and WG1/OsGRX8-dependent regulation of the oligomerization of the atypical Gγ subunit GS3, resulted in increased grain length [59].

The binding of apoplastic glucose modifies the functions of the regulator of G protein signalling 1 (RGS1) in tomato, triggering RGS1 endocytosis and uncoupling of the RGS1-Gα (GPA1) and GPA1-Gβ (SlGB1) proteins. In this way, the low-light-induced susceptibility of tomato to *P. syringae* pv *tomato* (*Pst*) DC3000 was alleviated by exogenous glucose treatment [60]. Moreover, the sensitivity of tomato plants to heat stress was decreased under high CO_2_, an effect that was linked to increases in apoplastic glucose levels [38]. High CO_2_-dependent regulation of thermotolerance was found to be mediated by RGS1. Elevated CO_2_- and high temperature-induced increases in apoplastic glucose accumulation trigger RGS1 endocytosis and the subsequent dissociation of RGS1-GPA1, releasing free GPA1 and regulating heat tolerance [38]. Taken together, such findings demonstrate that apoplastic sugar signalling and redox regulation of G protein functions modulate developmental and defence processes. Glucose signalling is important in the control of growth processes such as the de-etiolation of seedlings upon light exposure following seed germination. This signalling pathway involves hexokinase 1 and growth regulator 5, which is a transcriptional regulator of growth in *Arabidopsis* that binds to the promoter of the gene encoding phytochrome A (phyA). Hexokinase 1 operates both as a glycolytic enzyme and a glucose-activated sensor that triggers a decrease in phytochrome A levels. Glucose accumulation in dark-grown etiolated *Arabidopsis* cotyledons was found to stimulate the hexokinase 1-dependent increase in growth regulator 5, leading to a decrease of phyA and de-etiolation of seedlings upon light perception [61]. It will be interesting to determine whether eCO_2_ influences this type of developmental regulation.

Sugar transporters belonging to the sucrose transporter/carrier (SUT/SUC) family and the SUGARS WILL EVENTUALLY BE EXPORTED TRANSPORTER (SWEET) sugar carriers not only play important roles in carbohydrate partitioning in different plant tissues including leaves, flowers and seeds but also in the control of plant immune responses [62]. Impairment in the activities of the principal SWEETs and SUTs involved in apoplastic sucrose phloem loading leads to the accumulation of carbohydrates in the leaves, reduced photosynthesis and poor growth [63,64]. The bacterial and fungal pathogens of rice activate the expression of SWEET genes to gain access to the nutrients required to support virulence. The overexpression of SWEETs in rice results in lower growth and yield but enhances disease resistance and stress tolerance. For example, the co‐overexpression of the rice OsSUT1, OsSWEET11a and OsSWEET14 genes reduced sucrose synthesis and transport but increased the pathogen-induced HR leading to decreased susceptibility to infection by *Xanthomonas oryzae* pv. *oryzae* [65]. Little is known about the effect of eCO_2_ on the expression of SWEET genes or whether the results obtained with rice in response to *Xanthomonas* extend to other species, but such results clearly suggest the existence of interactions between the extent of apoplast sugar accumulation, ROS production and plant defences that are related to the signalling pathways of pathogen nutrition.

## 4. CO_2_ signalling regulates plant/microbe interactions and immunity

Growth under high CO_2_ exerts many local and systemic effects on plants, including changes in redox homeostasis, hormone signalling, root development and defence responses [23,66,67]. The eCO_2_-dependent increase in ROS is related to increases in the expression of *AtRBOHD* and *AtRBOHF* [67]. The increase in salt tolerance observed in tomato plants under eCO_2_ was linked to RBOH-dependent ROS production by NADPH oxidases [68]. Accumulating evidence provides support for the concept that eCO_2_-driven, ROS-dependent signalling pathways drive processes as diverse as stomatal closure [12], photosynthetic acclimation [69], mycorrhizal symbiosis and root phosphate uptake [70] and innate immune resistance to pathogens and herbivores. Within this context, eCO_2_-dependent ROS accumulation plays a central role in priming stress defences [71,72]. Further research is, however, required to determine the extent to which sugar signalling, particularly in the apoplast, participates in the orchestration of such responses. Regardless, it is possible to speculate that the presence of both sugar-dependent and sugar-independent eCO_2_ signalling pathways, that are not substantially overlapping, must confer a physiological advantage in a dynamic and often hostile environment.

### (a) Salicylic acid and jasmonic acid defence pathways

Growth under eCO_2_ generally enhances plant resistance to biotrophs or hemibiotrophs (such as some viruses and bacteria) [73–75] but weakens resistance to necrotrophs [76,77]. However, the extent to which eCO_2_ influences immunity depends upon the plant species and the type of pathogen involved, as well as the methods of CO_2_ enrichment [78]. Considerable research has focused on whether eCO_2_ activates plant defence hormones that are important in plant immunity, particularly salicylic acid (SA) and jasmonic acid (JA), with the former typically being important in resistance to biotrophs or hemibiotrophs, and the latter typically being required for immunity to necrotrophs [79–81]. Nevertheless, very complex changes in immune responses can be observed under eCO_2_ conditions. For example, growth under eCO_2_ improved shoot growth in *Brachypodium distachyon* and enhanced the expression of genes encoding proteins involved in plant immunity and secondary metabolism, particularly the terpenoid and phenylpropanoid pathways. However, the *B. distachyon* plants showed an enhanced susceptibility to the fungus *Magnaporthe oryzae* under these conditions but with no change in fungal development [82].

Pathogen-induced increases in SA accumulation and the expression of genes in downstream signalling pathways, e.g. encoding pathogenesis-resistance (PR) proteins, have been reported to increase under elevated CO_2_ compared to ambient CO_2_ conditions in many plant species, including tomato, tobacco and soybean [76,83,84]. The activation of the SA pathway by elevated CO_2_ has been suggested to be independent of the pathogen-induced pathway and to have a negative effect on plant growth [67]. Interestingly, a depletion of the antioxidant glutathione or impaired RBOH enzyme functions blocked CO_2_-induced SA-dependent defences, suggesting that eCO_2_-induced activation of SA signalling is redox-dependent [67]. Moreover, chloroplast carbonic anhydrases (CAs) such as SABP3 are SA-binding proteins [85]. The nitrosylation of AtSABP3 inhibits both CA activity and SA-binding capacity [86].

JA signalling pathways may also be modified under eCO_2_ because of inhibition of the lipoxygenase (LOX) pathway, which is important in JA synthesis. The eCO_2-_mediated suppression of JA-dependent signalling resulted in increased susceptibility to herbivorous insects in soybean, *Botrytis cinerea* in tomato and *Colletotrichum gloeosporioides* in tea [76,77,87]. In contrast, growth under eCO_2_ led to an increase in JA content in *Arabidopsis*, accompanied by enhanced resistance to fungal diseases [67]. Taken together, these results suggest that the influence of eCO_2_ on JA signalling may be highly dependent on the plant and pathogen species under investigation.

### (b) Carbonic anhydrases

**Figure 3.**
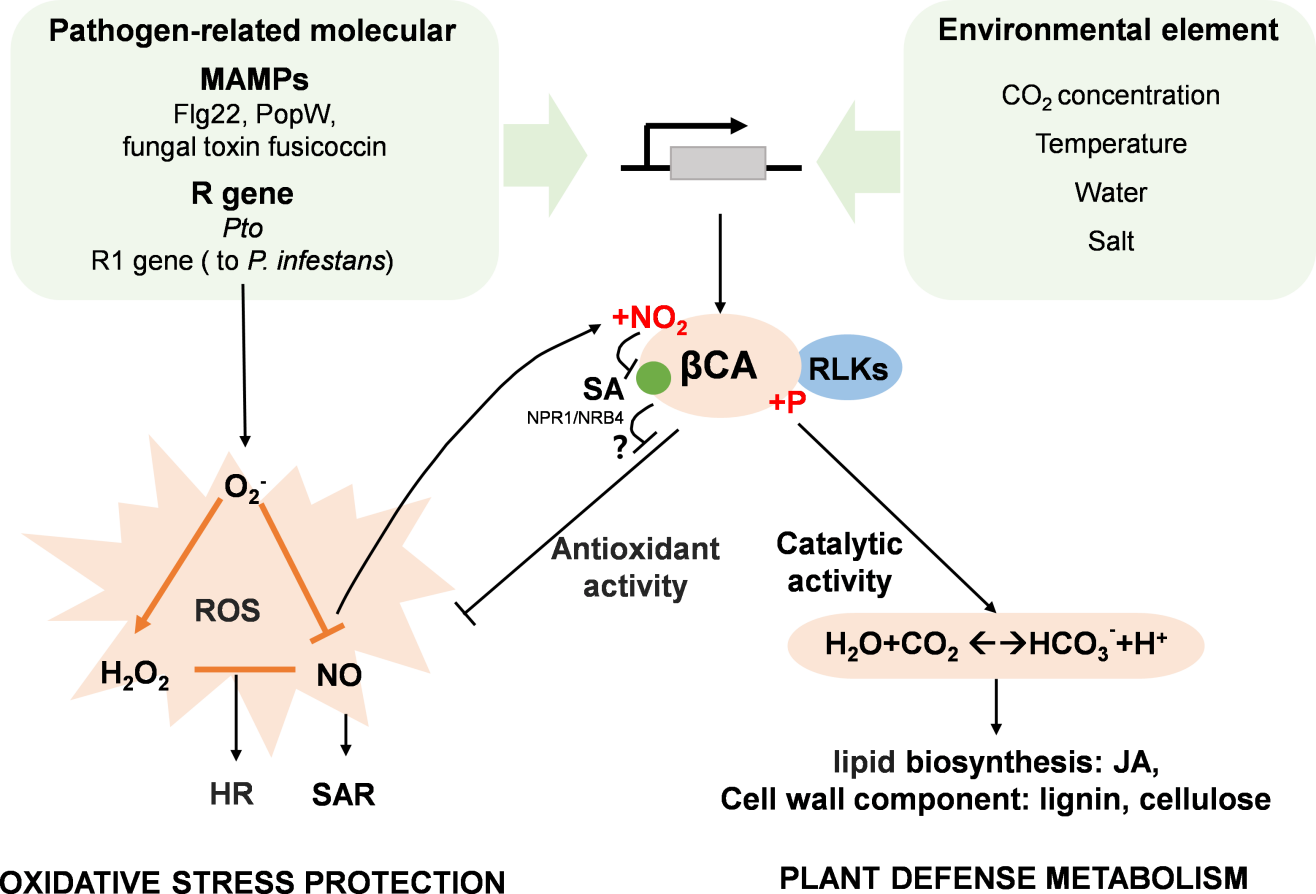
The central role of the β−carbonic anhydrases in CO_2_ signalling in plant defence. βCA, βcarbonic anhydrase; Flg22, 22 amino acid flagellin peptide, JA: jasmonic acid; MAMPs, microbe-associated molecular patterns; NO, nitric oxide; R, resistance; ROS, reactive oxygen species; MAPK, Mitogen activated protein kinase; RLK, receptor-like kinases; SA, salicylic acid; SAR, systemic acquired resistance.

Carbonic anhydrases (CAs) catalyze the reversible hydration of carbon dioxide into bicarbonate (HCO_3_^-^) and protons. In humans, these zinc-containing metalloenzymes can act as antioxidants in cells that are experiencing oxidative damage [88]. In plants, CAs function as intracellular CO_2_-sensing and regulating proteins [89] that link ambient CO_2_ levels to the hypersensitive response (HR) and immunity [85,90]. Higher plants contain three distinct CA families, αCAs, βCAs and γCAs, and each family is represented by multiple isoforms [91]. The dominant CA type in photosynthetic organisms, βCA, plays an important role in resistance responses to a wide range of biotic stresses (figure 3). The tobacco NbβCA1 and the *Arabidopsis* AtβCA1 forms, which are abundant in chloroplasts, are SA-binding proteins that induce defence responses to pathogens [85,86]. AvrPto-induced HR was not detected in the *βca1* mutant [85]. The extent and duration of HR depends on a balance between superoxide (O_2_^-^), H_2_O_2_ and NO levels [92]. Precisely how CAs are involved in this complex regulation is unknown. Interestingly, the interaction between CA and SA is inhibited by *S*-nitrosylation of the CA protein [86].

Moreover, the balance between bicarbonate and CO_2_ regulates many resistance-related metabolic pathways, particularly carbon-based secondary metabolites and JA-related pathways [93,94]. For example, the phosphorylation modifications of tomato SlβCA3 by a RLK is required for regulating cell wall metabolism and mediated eCO_2_-induced resistance to *Pst* [95].

### (c) Reactive oxygen species homeostasis

The defence-associated metabolism observed in *Arabidopsis catalase 2* (*CAT*) mutants in response to pathogens was modified in double mutants that also lacked *RBOHD* or *RBOHF* [96]. While the effect was most marked in *cat2* mutants that also lacked the AtRbohF protein [96], such findings suggest a level of co-operation between the apoplastic and intracellular redox signalling systems in plant responses to pathogens. There is extensive communication between the ROS-producing and processing systems between the apoplast and the cytosol and between the different organelles [97]. For example, the movement of ROS from the apoplast to the cytosol is essential for the propagation of calcium signals that is required for cell-to-cell signalling and plant stress acclimation. This process involves the calcium-permeable channel MECHANOSENSITIVE ION CHANNEL LIKE 3, CALCINEURIN B-LIKE CALCIUM SENSOR 4 (CBL4), CBL4-INTERACTING PROTEIN KINASE 26 and the Sucrose-nonfermenting−1-related protein kinase 2.6 called OPEN STOMATA 1 [44]. Moreover, there is an extensive exchange of reducing equivalents and pyridine nucleotides that involves shuttling of metabolites between the chloroplasts, mitochondria and peroxisomes during photosynthesis and photorespiration. Systems such as malate/oxaloacetate antiporters and the malate dehydrogenase (MDH)-associated malate valve [98,99], play important roles in adjusting stromal ATP/NADPH ratios and maintaining chloroplast redox homeostasis under changing environmental conditions. These systems also participate in signal integration and acclimation of plants to high CO_2_ [100]. The chloroplast and mitochondrial MDHs also play important roles in the regulation of ROS homeostasis, which has been linked to the regulation of programmed cell death [101]. Transporters such as the chloroplast inner membrane triose-P transporter also regulate stress responses through MAPK-dependent pathways [102].

## 5. High CO_2_ effects on the nutritional quality of food

An often-overlooked aspect of eCO_2_ and climate change is the negative impact on the nutritional quality of plant-based foods. It has long been recognized that growth under eCO_2_ increases organ carbohydrate availability, increasing C:N ratios and decreasing leaf nitrogen [103,104]. There is little doubt that eCO_2_ causes a general shift to a carbon-rich, nutrient poor composition of seeds and vegetables. Accumulating literature evidence supports the conclusion that eCO_2_ decreases the protein and mineral contents of plant-based foods [4,105–107], a consequence that has dire global consequences for human and animal nutrition and health.

While the adverse effects of eCO_2_ on plant protein and mineral levels are widely recognized, the mechanistic basis for this response is poorly understood. While a dilution effect caused by the eCO_2_-dependent stimulation of growth is often cited as the basis for the observed deterioration in nutritional quality, the metabolic and molecular basis for this regulation is not known and is poorly documented in the literature. There is likely to be a major genetic component to the eCO_2_-induced changes in nutritional contents, as shown, for example, by the inherent genetic variability in maintaining Zn level under eCO_2_ [5].

The decrease in chloroplast Pi levels that occurs in plants grown under eCO_2_ regulates the accumulation of phytic acid (inositol hexaphosphate). This sugar-phosphate metabolite is important because its presence in plant foods decreases the bioavailability of essential nutrients such as Fe^3+^ and Zn in the human diet. The *Arabidopsis* transporter PHT4;3, which is expressed under eCO_2_, facilitates a regulated decrease in chloroplast Pi and phytate levels [108]. Manipulation of the expression of PHT4;3 demonstrated that the accumulation of Pi and phytate in chloroplasts under eCO_2_ has a negative impact on plant growth. The PHT4;3-mediated decrease in Pi transport into the chloroplasts under eCO_2_ led to decreased phytate synthesis, suggesting that this process is important in sustaining optimal growth [108].

Phytate is produced through stepwise phosphorylation of myo-inositol. This pathway is linked to the synthesis of the antioxidant, ascorbic acid, which is itself involved in iron uptake and storage. Ascorbate reduces Fe^3+^ suggesting that it could have a physiologically significant role in Fe uptake. In addition, ascorbate efflux plays a role in Fe^3+^ reduction in plants that use an Fe^2+^ uptake system involving ferric chelate reductase and an iron transporter. In dicotyledonous plants such as *Arabidopsis* and pea, iron is transported to the developing embryos in the seeds as complexes with citrate and malate (Fe(III)_3_Cit_2_Mal_2_, Fe(III)_3_Cit_3_Mal_1_, Fe(III)Cit_2_) [109]. Ascorbate efflux from the embryos reduces Fe(III) from the citrate-malate complexes, allowing Fe(II) uptake. The embryos of ascorbate-deficient *vtc2–4* and *vtc5 A. thaliana* mutants showed a lower Fe^3+^ reducing capacity and a 75% decrease in seed Fe concentration [109]. Moreover, one of the gulono-1,4 γ-lactone oxidases (GULLO) that is involved in ascorbate synthesis, GULLO2, was found to facilitate iron transport from the endosperm into developing *Arabidopsis* embryos [110].

The synthesis of ascorbate is linked to the phytate metabolism through the enzyme *myo*‐inositol oxygenase (MIOX). MIOX4 was expressed in the leaves and flowers of *A. thaliana* [111]. Overexpression in *Arabidopsis* resulted in a two- to threefold increase in ascorbate accumulation [111]. These findings suggested that *myo*‐inositol oxygenase could function in both the inositol and ascorbate pathways. The complete dephosphorylation of phytate releases *myo*‐inositol. The steady level of phytate is reduced in *Arabidopsis* lines overexpressing the phosphatase, while ascorbate was increased [112]. Moreover, the fruit-specific expression of a bacterial pyrophosphorylase, which hydrolyses inorganic pyrophosphate to Pi, resulted in increased fruit sugar and ascorbate levels [113]. Interestingly, apoplast hexose/sucrose ratios were modified in tomato plants with decreased levels of ascorbate oxidase, an enzyme that plays a key role in regulating the redox state in the apoplast [114]. However, much remains to be understood about the redox regulation of nutrient accumulation in plant organs and the impact of eCO_2_ on the pathways that regulate nutrient acquisition and storage in plant organs.

## 6. Conclusions and perspectives

The perception of eCO_2_ triggers an RBOH-dependent oxidative burst in the apoplast, which primes cell-to-cell signalling through compartment-specific modifications in redox processes, and which has important consequences for whole plant functions. For example, the redox-auxin-strigolactone systemic signalling cascade facilitates mycorrhizal symbiosis and phosphate uptake in tomato [70]. The perception of eCO_2_ in tomato shoots causes an RBOH1-dependent increase in IAA accumulation. Suppression of the tomato RBOH1 protein prevents CO_2_-induced accumulation of IAA in the shoot and inhibits the subsequent signalling to the roots that results in enhanced mycorrhizal symbiosis [70]. We have provided evidence in support of the concept that CO_2_ acts as an environmental signal as well as a substrate in plants. Control of oxidation in the apoplast by regulated ROS production is important in cell-to-cell CO_2_ signalling, as demonstrated, for example, in the systemic CO_2_-induced redox-auxin-strigolactone signalling cascade that has been characterized in tomato. This long-distance signalling pathway from leaves via the stem to roots ultimately promotes root growth and mycorrhizal associations and enhances phosphate uptake [48]. Auxin is also a trigger for ROS and NO accumulation, as demonstrated in the shade-induced *S*-nitrosylation of CONSTITUTIVELY PHOTOMORPHOGENIC 1 (COP1) to promote hypocotyl growth [115]. Similarly, eCO_2_ induced NO accumulation, which promotes root growth. Increased carbohydrate synthesis under elevated CO_2_ activates auxin- and ethylene-responsive signalling pathways, which in turn stimulate intracellular NO accumulation, inducing root hair growth through Ca^2+^ signalling and MAP kinase cascades [116–119].

**Figure 4.**
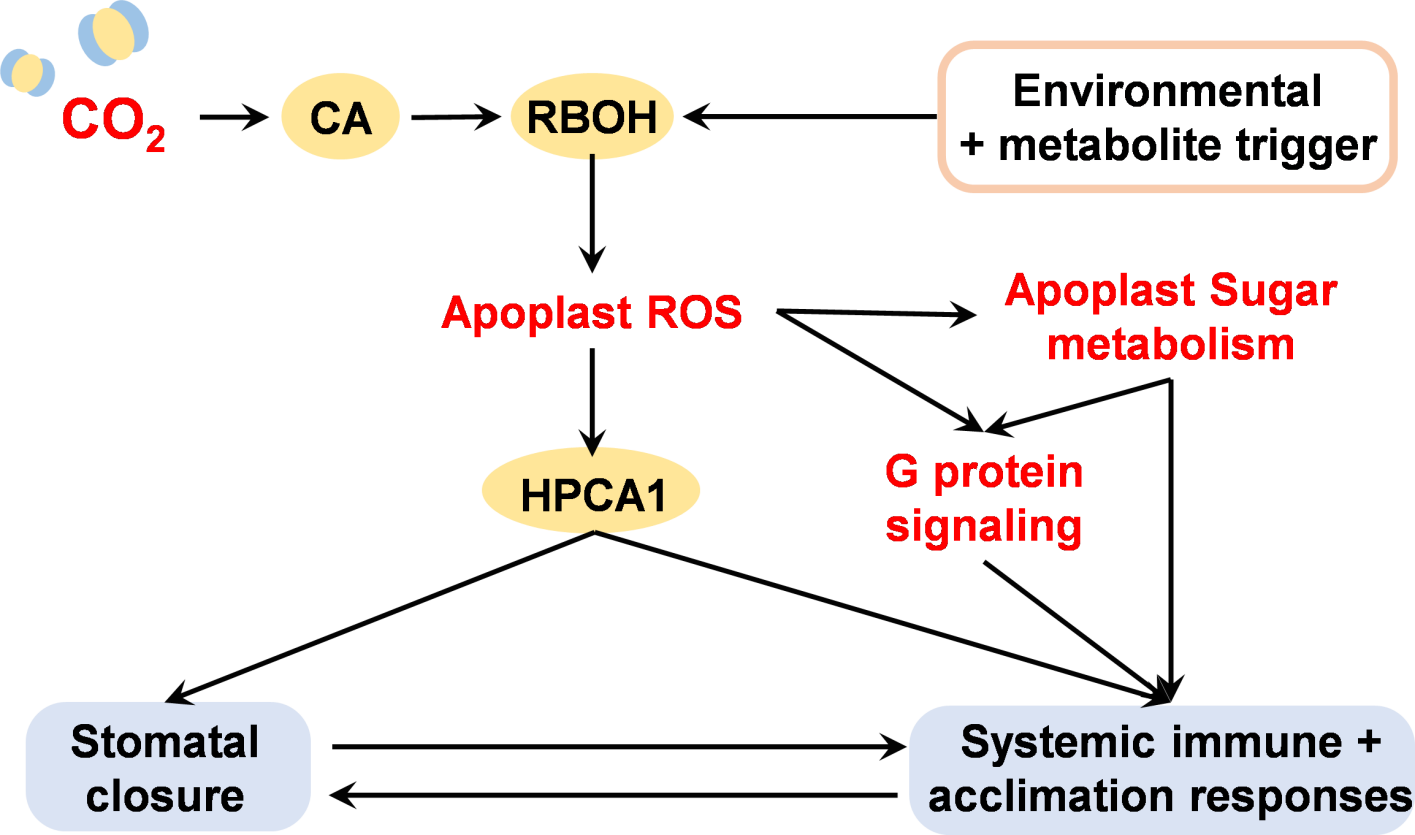
The H_2_O_2_ receptor HPCA1 links the regulation of guard cell closure and systemic cell-to-cell signalling in response to environmental perturbations. CA, carbonic anhydrase; HPCA1, Hydrogen peroxide Ca^2+^ increases 1; RBOH, respiratory burst oxidase homolog; ROS, reactive oxygen species.

We have summarized the evidence demonstrating the presence of CO_2_ receptors that trigger pathways that are independent of photosynthesis in order to regulate stomatal functions and activate systemic plant innate immune and defence responses. The RBOH-dependent oxidation of the apoplast is intrinsically involved in both eCO_2_-dependent stomatal closure and stress-induced cell-to-cell signalling. It is not a coincidence that the H_2_O_2_ receptor HPCA1 is required for both processes (figure 4). Since this receptor also acts as a quinone sensor, it is likely that changes in both apoplastic ROS and quinones trigger local and cell-to-cell signalling in response to stress perception. Further research is required to determine the relative importance of each in the elucidation of appropriate defence responses and whether these signalling molecules act synergistically.

We, therefore, postulate that the CO_2_ sensing and signalling pathways that operate in guard cells encompass and trigger ROS wave systemic signalling. While it is not yet clear whether all components identified in studies of stomatal signalling are involved in biotic defence response, there is an intriguing convergence at the level of the CO_2_-sensing βCA, as well as at HPCA1. The eCO_2_-dependent increase in the oxidation of the apoplast will also have an impact on sugar signalling through processes such as activation of G protein functions and altered sugar uptake.

Plants grown under eCO_2_ have an increased antioxidant capacity [120,121]. This acclimation response may serve to decrease the intensity or duration of RBOH-mediated ROS wave signalling. For example, mutants defective in APX show enhanced ROS wave signalling and systemic responses [122]. In addition, the inhibition of photorespiration under elevated CO_2_ decreases intracellular H_2_O_2_ production, and thus changes relative ROS production in the different compartments, with lower levels in peroxisomes and chloroplasts relative to the mitochondria and apoplast [11].

Future research is required to determine the extent to which CO_2_ signalling in guard cells is linked to ROS wave systemic signalling, as well as the proteins that are involved in signal transmission. A greater understanding of the complex ROS-dependent and ROS-independent pathways of CO_2_ signalling is required in order to address the issue of the negative impact of eCO_2_ on nutrient acquisition and storage in plant organs that serve as animal and plant food.

## Data Availability

This article has no additional data.
